# Prophylaxis of esophageal stricture after endoscopic submucosal dissection with autologous adipose-derived stromal cells

**DOI:** 10.1055/a-2868-3232

**Published:** 2026-07-23

**Authors:** Ylanna Fortes, Renata Nobre, Bruno da Costa Martins, Luciano Lenz, Adriana Vaz Safatle-Ribeiro, Cristina Pires Camargo, Fauze Maluf-Filho

**Affiliations:** 1 Endoscopy DepartmentCancer Institute of the State of São PauloSão PauloBrazil; 2 Plastic Surgery Department 28133 University of São PauloSão PauloBrazil

Esophageal endoscopic submucosal dissection (ESD) involving >75% of the circumference is associated with a stricture in >70–90% of cases. This impairs quality of life and limits ESD indications for superficial squamous cell carcinoma (SCC). Prophylactic corticosteroids have partially addressed this problem 1 2 . Recently, adipose tissue-derived stromal cell (ADSC) injection has emerged from animal trials. The advantages of ADSC are their paracrine activity, local immunomodulation, ability to differentiate into mesenchymal and non-mesenchymal lineages, and modulation of keratinocyte–fibroblast interaction, reducing excessive fibrosis development 3 4 5 .

**Fig. 1:**
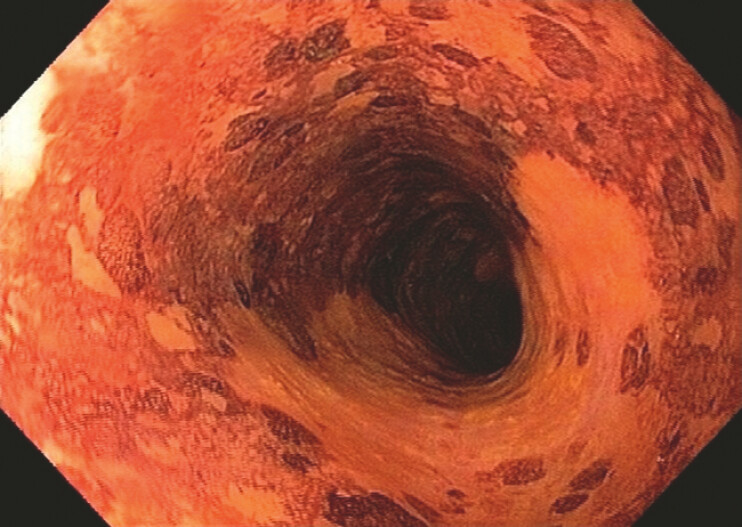
An 8cm-long lesion occupying 80% of the esophageal circumference.

**Fig. 2:**
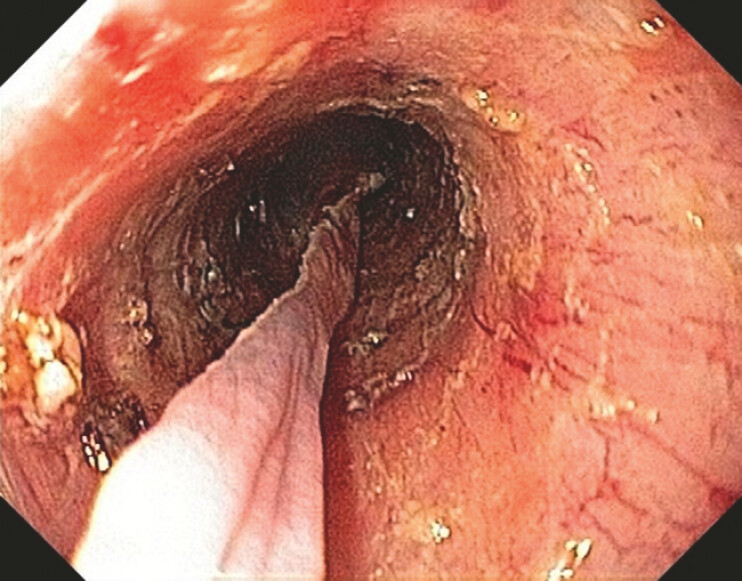
The final appearance after 90% of esophageal circumference ESD. ESD, endoscopic submucosal dissection.

**Fig. 3:**
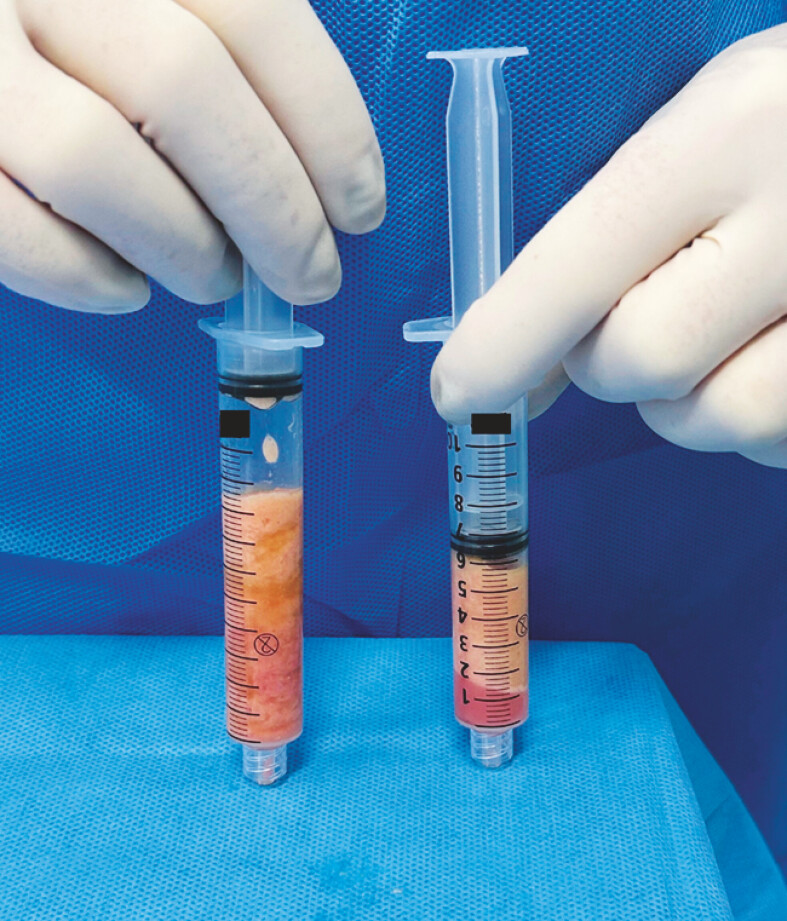
The Liposuction’s initial product on decantation.

**Fig. 4:**
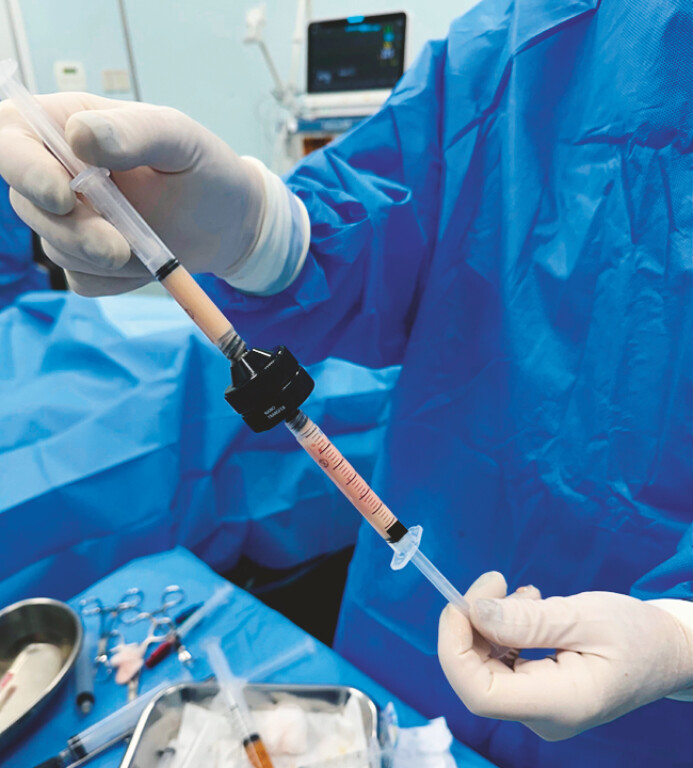
Fat fragmentation.

We report a novel prophylactic strategy using submucosal injection of mechanically processed autologous ADSC into the ESD ulcer bed. A 71-year-old male patient, diagnosed with SCC, occupying 80% of luminal circumference ( Fig. 1 ) underwent a 90% of esophageal circumference, 8 cm-long ESD ( Fig. 2 ). Once ESD was finished, a plastic surgeon made a liposuction using two 10 mL syringes ( Fig. 3 ). The fat was fractioned ( Fig. 4 ) and 15 mL were injected onto the ESD resection bed submucosa, through a 5Fr catheter. Oral prednisone was also administered. Pathology revealed early squamous cell carcinoma (pT1a–M2), without angiolymphatic invasion, with free margins. Serial endoscopies at 2, 3, 4, 5, 6, 8 and 12 weeks showed progressive healing. At week 8, the ulcer was completely healed with cicatricial retraction. The patient developed transitory dysphagia that completely resolved. In none of the endoscopies, lumen narrowing was observed, and endoscopic dilation was not necessary. The patient remains clinically well, without dysphagia or weight loss, 5 months after the procedure ( Video 1 ).

Autologous adipose-derived stromal cell injection on the ESD ulcer bed after a 90% of esophageal circumference resection for stricture prophylaxis. ESD, endoscopic submucosal dissection.Video 1

This first experience shows the feasibility and promising efficacy of immediate autologous ADSC injection for preventing the stricture after high-risk esophageal ESD, with rapid processing and no added adverse events. This strategy may improve extensive esophageal resection prognosis. Further multicenter trials are essential to establish its role in extensive circumferential ESD.

Endoscopy_UCTN_Code_TTT_1AO_2AH
